# Efficacy, Immunogenicity, and Safety of the Two-Dose Schedules of TURKOVAC versus CoronaVac in Healthy Subjects: A Randomized, Observer-Blinded, Non-Inferiority Phase III Trial

**DOI:** 10.3390/vaccines10111865

**Published:** 2022-11-04

**Authors:** Mine Durusu Tanriover, Ozlem Altuntas Aydin, Rahmet Guner, Orhan Yildiz, Ilhami Celik, Hamdi Levent Doganay, Sukran Kose, Sila Akhan, Emin Halis Akalin, Zafer Sezer, Aykut Ozdarendeli, Serhat Unal, on behalf of the TURKOVAC Study Group

**Affiliations:** 1 Department of Internal Medicine, Hacettepe University Faculty of Medicine, 06230 Ankara, Türkiye; 2 Vaccine Institute, Hacettepe University, 06230 Ankara, Türkiye; 3 Department of Infectious Diseases and Clinical Microbiology, University of Health Sciences, Başaksehir Cam and Sakura City Hospital, 34480 Istanbul, Türkiye; 4 Infectious Diseases and Clinical Microbiology Clinic, Ankara Yildirim Beyazit University, Ankara City Hospital, 06800 Ankara, Türkiye; 5 Department of Infectious Diseases and Clinical Microbiology, Erciyes University Faculty of Medicine, 38030 Kayseri, Türkiye; 6 Department of Infectious Diseases and Clinical Microbiology, Kayseri City Training and Research Hospital, 38080 Kayseri, Türkiye; 7 Department of Gastroenterology, Medical Park Pendik Hospital, 34899 Istanbul, Türkiye; 8 Department of Internal Medicine, Bahcesehir University School of Medicine, 34734 Istanbul, Türkiye; 9 Infectious Diseases Clinic, University of Health Sciences, Izmir Tepecik Training and Research Hospital, 35020 Izmir, Türkiye; 10 Department of Infectious Diseases and Clinical Microbiology, Kocaeli University Faculty of Medicine, 41001 Kocaeli, Türkiye; 11 Department of Infectious Diseases and Clinical Microbiology, Bursa Uludag University Faculty of Medicine, 16059 Bursa, Türkiye; 12 Department of Medical Pharmacology, Erciyes University Faculty of Medicine, 38030 Kayseri, Türkiye; 13 Department of Microbiology, Erciyes University Faculty of Medicine, 38030 Kayseri, Türkiye; 14 Vaccine Research, Development and Application Centre (ERAGEM), Erciyes University, 38280 Kayseri, Türkiye; 15 Department of Infectious Diseases and Clinical Microbiology, Hacettepe University Faculty of Medicine, 06230 Ankara, Türkiye

**Keywords:** TURKOVAC, CoronaVac, efficacy, safety, COVID-19, vaccine, vaccination, vaccine immunogenicity, SARS-CoV-2 vaccines

## Abstract

We present the interim results of the efficacy, immunogenicity, and safety of the two-dose schedules of TURKOVAC versus CoronaVac. This was a randomized, observer-blinded, non-inferiority trial (NCT04942405). Volunteers were 18–55 years old and randomized at a 1:1 ratio to receive either TURKOVAC or CoronaVac at Day 0 and Day 28, both of which are 3 μg/0.5 mL of inactivated severe acute respiratory syndrome coronavirus-2 (SARS-CoV-2) adsorbed to aluminum hydroxide. The primary efficacy outcome was the prevention of polymerase chain reaction (PCR)-confirmed symptomatic coronavirus disease 2019 (COVID-19) at least 14 days after the second dose in the modified per-protocol (mPP) group. Safety analyses were performed in the modified intention-to-treat (mITT) group. Between 22 June 2021 and 7 January 2022, 1290 participants were randomized. The mITT group consisted of 915 participants, and the mPP group consisted of 732 participants. During a median follow-up of 90 (IQR 86–90) days, the relative risk reduction with TURKOVAC compared to CoronaVac was 41.03% (95% CI 12.95–60.06) for preventing PCR-confirmed symptomatic COVID-19. The incidences of adverse events (AEs) overall were 58.8% in TURKOVAC and 49.7% in CoronaVac arms (*p* = 0.006), with no fatalities or grade four AEs. TURKOVAC was non-inferior to CoronaVac in terms of efficacy and demonstrated a good safety and tolerability profile.

## 1. Introduction

Coronavirus disease 2019 (COVID-19) is keeping its pace through the newly emerging variants of severe acute respiratory syndrome coronavirus-2 (SARS-CoV-2) and affecting the globe in different dimensions. Additionally, the burden of disease in terms of COVID-19 and all-cause mortality is exceptionally high in underserved populations with social disparities, among people with underlying chronic conditions, and in low- and middle-income countries [1].

Vaccination is the crucial pillar in breaking the transmission chain of SARS-CoV-2 infections in combination with mask-wearing, social distancing measures, and indoor ventilation [2]. However, vaccine equity is an alarming issue given that 63.3% of the world population has received at least one dose of a COVID-19 vaccine, whereas only 13.6% of people in low-income countries have received at least one dose at the time this article has been drafted [3]. It is clear that the equitable and fair distribution of vaccines is crucial in order to end the pandemic, and the World Health Organization (WHO) has declared urgency to accelerate vaccinations in low- and middle-income countries [4]. It has been argued that COVID-19 vaccination strategies must focus on preventing severe disease, and for this purpose, at least one dose of COVID-19 vaccination coverage should be attained among adults globally [5].

As of 8 March 2022,there were 147 vaccine candidates in clinical trials, 21 (14%) of which are inactivated vaccines [6]. Inactivated vaccines have certain advantages, such as having a well-established production system and not requiring advanced transport and storage conditions other than a 2–8 °C cold chain, which make them valuable, especially for developing countries. Additionally, as they include a diversity of antigens that are more prone to be conserved than the S protein, such as the nucleocapsid, envelope, and matrix proteins, they offer additional antigenic targets, which might boost protection [7]. However, they require biosafety level-3 facilities and verification of the integrity of antigens and/or epitopes and adjuvants to enhance the immune response. Although the inactivated vaccines have a predictable and favorable long-term safety profile, decreased neutralization capacity has been demonstrated for variants of concern (VOC) [8].

The whole virion-inactivated vaccines on the WHO emergency use list are CoronaVac (Sinovac Life Sciences Co., Ltd., Beijing, China), Covaxin (Bharat Biotech International Ltd., Telangana, India), and Inactivated COVID-19 Vaccine (Vero Cell) (Beijing Institute of Biological Products Co., Ltd. [BIBP], Beijing, China) [9] and they make up nearly half of all vaccines administered throughout the world, with CoronaVac being the most widely administered [10]. The interim analysis of the phase III trial of CoronaVac in Türkiye revealed a vaccine efficacy of 83.5% (95% confidence interval [CI] 65.4–92.1; *p* < 0.0001) for symptomatic COVID-19 with no severe COVID-19 cases or deaths during the follow-up [11]. TURKOVAC (Koçak Farma Production Facilities, Tekirdağ, Türkiye) is also an inactivated whole virion vaccine developed with the SARS-CoV-2 strain hCoV-19/Türkiye/ERAGEM-001/2020 isolated from a patient in Türkiye with confirmed COVID-19 [12]. The preclinical immunogenicity, protective efficacy, and safety evaluation of TURKOVAC (formerly ERUCoV-VAC) were tested in BALB/c mice, transgenic mice (K18-hACE2), and ferrets, and no safety issues were observed, while the vaccine candidate induced humoral immune responses in old and young BALB/c mice, protected K18-hACE2 transgenic mice against a lethal SARS-CoV-2 challenge and reduced upper respiratory tract SARS-CoV-2 infection in ferrets [12]. The vaccine candidate was then introduced to phase I and II trials, where safety and immunogenicity analyses were performed with 3 μg/0.5 mL and 6 μg/0.5 mL TURKOVAC versus placebo. The phase I trial with a 21-day dosing schedule demonstrated that 84% of the vaccinated subjects exhibited neutralizing antibodies, which did not differ between the two administered vaccine doses [13]. Anti-SARS-CoV-2 specific antibodies were found in all Day 43 sera from vaccine-treated volunteers. The phase II trial, which was run with a 28-day dosing schedule, demonstrated that total immunoglobulin (Ig)G responses against SARS-CoV-2 by Enzyme-Linked ImmunoSorbent Assay (ELISA) were significantly higher in the 6 μg group compared to the 3 μg group; however, there was no significant difference between neutralizing antibody titers and T cell response determined by the ELISPOT [13]. TURKOVAC, at a dosage of 3 μg/0.5 mL in 28-day dosing intervals, was introduced to the phase III trial with CoronaVac as the active comparator to test the non-inferiority of TURKOVAC in terms of efficacy, immunogenicity, and safety.

Here, we present the interim results of the efficacy, immunogenicity, and safety of the two-dose schedules of TURKOVAC versus CoronaVac.

## 2. Materials and Methods

### 2.1. Trial Design

This is a randomized, observer-blinded, non-inferiority phase III clinical trial to assess the efficacy, immunogenicity, and safety of the two-dose TURKOVAC versus the two-dose CoronaVac among volunteers between 18–55 years old in Türkiye (Registered at ClinicalTrials.gov, NCT04942405). Enrolled participants were randomly assigned in a ratio of 1:1 to one of the two arms to receive either 0.5 mL of the inactivated study vaccine TURKOVAC (vaccine lot numbers: 9020103, 9020104, 9020106, 9020112, 9020120) or 0.5 mL of CoronaVac (vaccine lot number: MF2106047) to be administered as two doses 28 days apart. As the trial utilized a non-inferiority design with an active comparator, we aimed to include a comparator vaccine that would have a similar mode of action to compare the efficacy and also which was an authorized vaccine already in use and trusted by the community in Türkiye in order to increase the enrollment rate. Randomization was performed by the Interactive Web-based Response System (IWRS) of the Omega Clinical Research Organization (CRO), Ankara, Türkiye.

Participants were recruited in eight centers between 22 June 2021 and 7 January 2022. Initially, three centers were activated for safety follow-up until 400 volunteers were recruited, which was attained on 4 August 2021. Automated phone calls were made daily for the first enrolled 400 subjects to screen for adverse events (AEs) and COVID-19 symptoms until 21 days after the second dose. As per protocol, 21 days after the second vaccination of the 400th subject included in the study, the Data Safety Monitoring Board had a meeting and reported the safety results obtained until 21 September 2021. The report was submitted to the Ethics Committee, and the further recruitment of subjects was pursued after the decision that no major safety concern was raised. In the second phase, enrolment continued in eight centers.

The protocol of the trial was drafted when the alpha variant was the dominant VOC, and the incidence rates of COVID-19 were relatively low. However, after the commencement of the trial, the Delta variant became the dominant variant quickly, and the incidence rates raised dramatically. The sample size was recalculated based on the current disease incidence rate, and a protocol amendment was planned; however, the primary endpoint was reached on 10 November 2021. Moreover, on 22 December 2021, the Ministry of Health (MoH) gave an Emergency Use Authorization (EUA) for TURKOVAC and started its roll-out for the community vaccination program in the last week of December 2021.

The study protocol was approved by the Clinical Research Ethics Board of Hacettepe University (No: KA-21070 and Date: 21 June 2021).

### 2.2. Participants

Volunteers 18–55 years of age with no COVID-19 history were screened for eligibility. Those who consented to participate agreed to comply with all study visits, procedures, contraceptive requirements, and were medically stable, and were enrolled. Exclusion criteria included: acute illness or fever within 48 h before or use of antipyretic/analgesic medication within 24 h before planned administration of vaccine; pregnancy or breastfeeding; known history of SARS-CoV-2 infection; current positive (polymerase chain reaction (PCR)-based viral RNA detection) or past positive (serological testing or PCR-based viral RNA detection) diagnostic test result for SARS-CoV-2; prior administration of an investigational or approved coronavirus vaccine or current/planned simultaneous participation in another interventional study to prevent or treat COVID-19; cardiac diseases; uncontrolled hypertension; history of coronary artery disease at early ages in their first-degree relatives (presence of coronary artery disease before age 55 in men and before age 65 in women); body mass index ≥ 40 kg/m^2^; autoimmune disease; severe allergic reaction to any licensed or investigational vaccine or to any of the constituents of CoronaVac or TURKOVAC; bleeding disorders; immunosuppressive or immunodeficient state (including human immunodeficiency virus), asplenia, recurrent severe infections; receipt or plan of receipt of a licensed, live replicating vaccine within 28 days before or after first study vaccination or a licensed inactivated or non-replicating vaccine within 14 days before or after first study vaccination; immunosuppressive therapy within 6 months prior to screening, or planned receipt throughout the study; receipt of systemic Igs or blood products within 3 months prior to screening or plans to receive such products during the study.

### 2.3. Procedures

All participants had oropharyngeal and nasopharyngeal swabs for baseline PCR testing with Bio-Speedy^®^Direct RT-qPCR SARS-CoV-2 detection kit (Bioeksen, Türkiye) on Bio-Rad CFX96 TouchTM platform (Foster City, CA, USA) at Visit 1. Blood samples were collected from all participants in Visit 1 and preserved until the closing of the enrolment, when all of the samples were analyzed for the baseline SARS-CoV-2 IgG antibody. The SARS-CoV-2 IgG II Quant assay (Abbott Ireland Diagnostics Limited, Sligo, Ireland), a chemiluminescent microparticle immunoassay (CMIA) was used for the qualitative and quantitative determination of IgG antibodies to SARS-CoV-2 in human serum and plasma on the ARCHITECT i System. This assay utilizes a 4 Parameter Logistic Curve fit data reduction method (4PLC, Y-weighted) to generate a calibration and results. The cut-off is 50.0 AU/mL for the interpretation of results. Follow-up samples for SARS-CoV-2 anti-spike IgG were collected at least 14 days after the second dose of the vaccine. Simultaneous samples were collected for the pseudovirus neutralization test to check for neutralizing activity. For this test, the DIA.PRO ACE2-RBD neutralization assay kit (Diagnostic Bioprobes Srl, Sesto San Giovanni, Italy) was used. The sensitivity of this kit is reported to be better than 90% with reference to the gold-standard method of neutralization in vivo. In addition, the specificity of this assay is reported to be >98%.

Symptom-based active surveillance was performed to detect participants with symptoms suggesting COVID-19 during the follow-up (script—Supplementary Material S2). All cases of SARS-CoV-2 infection were classified according to the scale of clinical progression proposed by the WHO [14].

### 2.4. Outcomes and Analysis Methods

The primary objective was to evaluate the efficacy of a two-dose regimen of TURKOVAC and a two-dose regimen of CoronaVac for reverse transcription (RT)-PCR-confirmed symptomatic COVID-19 disease. The primary endpoint was the protection rates of two doses of TURKOVAC and two doses of CoronaVac against RT-PCR-confirmed symptomatic COVID-19 at least 14 days after the second vaccination dose.

For evaluating the efficacy of the study vaccines, COVID-19-free person-years were calculated for both study arms. Accordingly, the time from the anticipated date of prevention (14 days after the administration of the second dose) to either the date of data cut-off or the date of a PCR-confirmed diagnosis of COVID-19 was determined for each participant and summed to calculate the total person-years without the disease. Total person-years was divided by the number of participants diagnosed with COVID-19 to determine the vaccine efficacy in the CoronaVac and TURKOVAC groups.

The efficacy measure was defined as the disease events per person-years of COVID-19-free period, and the relative risk reduction was used to compare vaccine efficacies, which was calculated as below:

$$\mathrm{Primary}\;\mathrm{efficacy}\;\mathrm{measure}=\frac{\sum^{\;}\mathrm{Cases}}{\sum^{\;}\mathrm{Time}\;\mathrm{to}\;\mathrm{event}}$$

$$\begin{array}{l} \mathrm{Primary}\;\mathrm{efficacy}\;\mathrm{measure}\;\mathrm{(PEM)}:\;\mathrm{incidence}\;\mathrm{rate}\;\mathrm{of}\;\mathrm{symptomatic}\;\mathrm{COVID-19}\;\mathrm{cases}\\ \mathrm{Cases}:\;\mathrm{RT-PCR}\;\mathrm{confirmed}\;\mathrm{COVID-19}\;\mathrm{cases}\\ \mathrm{Time}\;\mathrm{to}\;\mathrm{event}:\;\mathrm{Time}\;\mathrm{from}\;\mathrm{vaccine}\;\mathrm{protection}\;\mathrm{to}\;\mathrm{diagnosis}\;\mathrm{or}\;\mathrm{unveiling}\;\mathrm{of}\;\mathrm{masking} \end{array}$$

$$\mathrm{Relative}\;\mathrm{Risk}\;\mathrm{Reduction}=100\times \left(1-\frac{{\mathrm{PEM}}_{\mathrm{TURKOVAC}}}{{\mathrm{PEM}}_{\mathrm{CoronaVac}}}\right)$$

Secondary objectives were:

- To evaluate the efficacy of a two-dose regimen of TURKOVAC and a two-dose regimen of CoronaVac for the prevention of hospitalization and death among RT-PCR-confirmed severe COVID-19 cases;
- To evaluate the efficacy of the first dose of TURKOVAC and the first dose of CoronaVac against RT-PCR-confirmed symptomatic COVID-19;
- To assess the safety of TURKOVAC and CoronaVac by determining the incidence of adverse reactions and serious AEs;
- To assess the immunogenicity of a two-dose regimen of TURKOVAC and a two-dose regimen of CoronaVac.

All AEs were questioned during all visits and through automated phone calls via the Interactive Voice Response System (IVRS) (script—Supplementary Material S2). Predefined symptoms (solicited events) and other unspecified symptoms (unsolicited events) reported by the participants were recorded. All safety data, until the date of data cut-off, were recorded and analyzed in the current report.

### 2.5. Statistical Analysis

It was planned to include 40,800 subjects in the study in the initial protocol. Assuming the incidence of confirmed COVID-19 (RT-PCR confirmed COVID-19 cases per 1000 person × year) to be 22 per 1000 person-year in the CoronaVac arm and 20 per 1000 person-year in the TURKOVAC arm and based on the use of a two-sided test at the alpha = 0.05 level of significance, a sample size of 18,546 participants per CoronaVac arm would provide 80% power to reject the null hypothesis. The non-inferiority margin was assumed as 10%, and the value was 0.0017%. The planned sample size was 40,800 participants when a dropout rate of approximately 10% was considered (20,400 subjects for the TURKOVAC arm and 20,400 subjects for the CoronaVac arm). The interim analysis would be performed when 20 cases were confirmed, and the final analysis would be performed when 40 cases of confirmed COVID-19 were reported. This calculation was done depending on the rate of symptomatic COVID-19 cases in the CoronaVac phase III study and an extrapolation of the expected symptomatic cases among the targeted sample size in the period where this study was planned. After the recruitment of the first 400 subjects in the first phase, along with the interim safety analysis, the sample size would be calculated again. However, as the protocol amendment and approval process were ongoing, the primary endpoint was reached, and TURKOVAC was granted EUA by the MoH.

As per protocol, blood samples for SARS-CoV-2 antibody detection were collected in Visit 1, and volunteers were enrolled and vaccinated after negative PCR test results were available. SARS-CoV-2 antibody test results were available after the study was closed to the enrolment of new subjects, and after the exclusion of those volunteers found to be seropositive at baseline, the following analysis sets were established for statistical analyses. The modified intention-to-treat (mITT) analysis set included all participants who received at least one dose of the study vaccine. The mITT set also formed the safety analysis set. The follow-up period for safety analyses was defined as the time period (days) from the randomization date to the data cut-off date, which was 23 February 2022. The modified per-protocol (mPP) analysis set included all eligible randomized participants who received two doses of TURKOVAC or two doses of CoronaVac within the predefined window in the protocol, had no evidence of current or prior SARS-CoV-2 infection at baseline or within 14 days after the second study vaccination, had no protocol deviations to affect efficacy and safety assessment. The follow-up period for efficacy analyses in the mPP set was defined as 90 days after 14 days after the second dose of the vaccine or the data cut-off date (23 February 2022), whichever came first.

All analyses were performed using IBM SPSS Statistics for Windows, Version 25.0 (IBM Corp., Armonk, NY, USA). Incidence of COVID-19 was calculated as the total number of episodes (lab-confirmed symptomatic COVID-19) person-years for the at-risk population * 1000. This was done according to per-protocol (PP) analysis. Demographic characteristics were summarized as descriptive. The normality of data was tested using the visual (histogram and probability plots) and analytical (Kolmogorov-Smirnov/Shapiro-Wilk tests) methods. Descriptive statistics were expressed as mean, standard deviation, and interquartile range (IQR) for numerical variables; categorical variables were expressed as numbers and percentages. The chi-square or Fisher’s exact test was used to compare proportions between groups. A Log-rank test was employed for the comparison of follow-up duration between the treatment arms.

The 95% CI for vaccine efficacy with the use of a binomial distribution-based exact method was calculated. The time to diagnosis of COVID-19 from the time of anticipated vaccine protection in both groups was presented using the Kaplan-Meier survival curves. An mPP analysis was used as a means of adjusting for the expected treatment-effect-by-baseline covariate interaction that would be present in a mITT analysis. By using an mPP analysis, the zero expected vaccine efficacy subgroup was eliminated, thus approaching an unbiased estimate of prophylactic vaccine efficacy.

Safety and efficacy analyses were performed in the mITT and in the mPP groups, respectively.

## 3. Results

### 3.1. Participants

**Figure 1:**
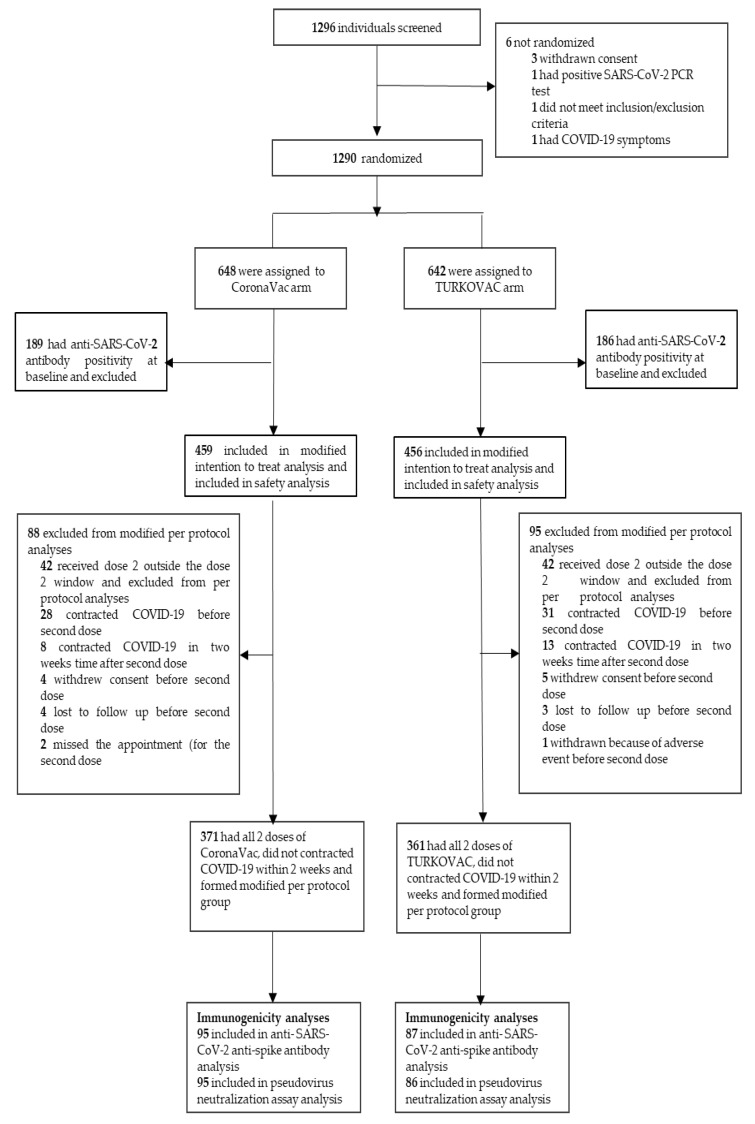
Study flowchart. SARS-CoV-2, severe acute respiratory syndrome coronavirus-2; PCR, polymerase chain reaction; COVID-19, Coronavirus disease 2019.

All recruitment, randomization, and follow-up procedures were completed in eight study centers (Supplementary Material S4). A total of 1296 volunteers were screened for eligibility, and 1290 were randomized between 22 June 2021 and 7 January 2022 (Figure 1).

Among randomized participants, 648 received CoronaVac, and 642 received TURKOVAC. After the exclusion of those who had positive baseline anti-SARS-CoV-2 antibodies, 459 participants in the CoronaVac arm and 456 participants in the TURKOVAC arm formed the mITT group. The mPP group included 371 participants in the CoronaVac arm and 361 participants in the TURKOVAC arm for primary efficacy analysis. On the date of data cut-off, 915 participants in the mITT group reached 132 (IQR 89–133) days of median follow-up after the first dose. The median age of the participants was 38 (IQR 33–44) years, and 665 (72.7%) were male. One hundred and forty-three participants (15.6%) reported at least one preexisting condition, with allergic conditions being the most prevalent ones. The main baseline characteristics of participants are given in Table 1.

### 3.2. Efficacy

**Figure 2:**
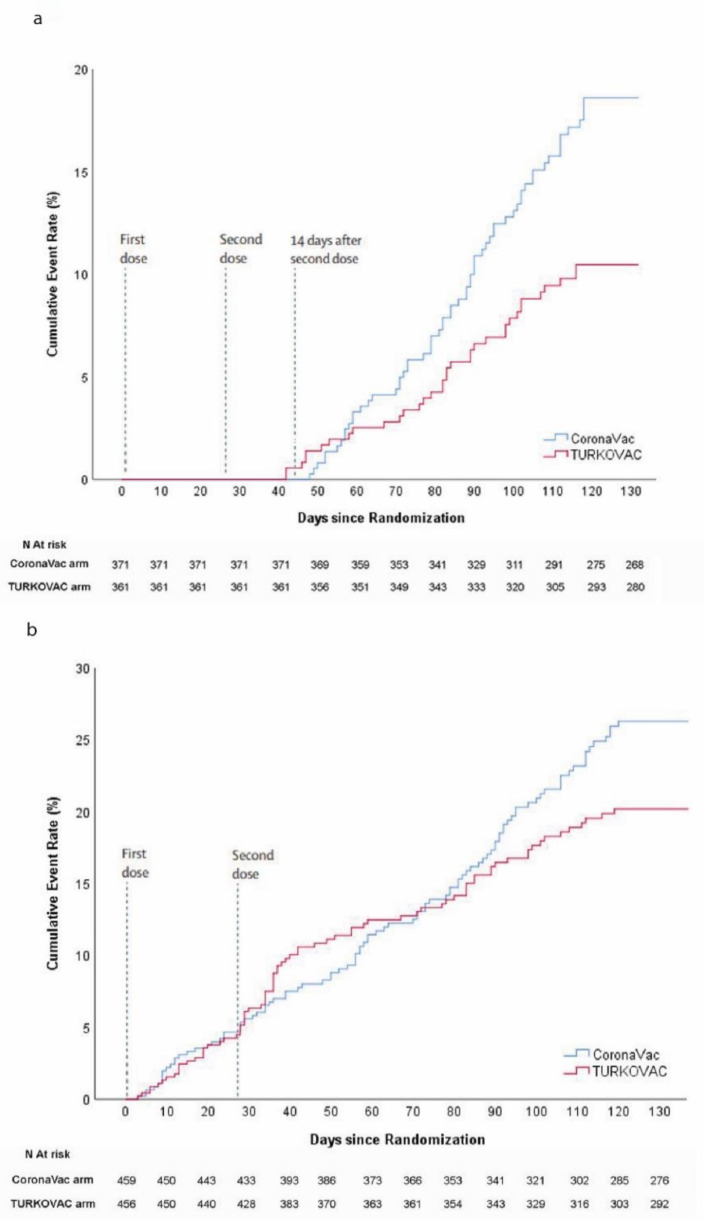
Cumulative incidence curves for COVID-19 cases in both study arms: (**a**) in the modified per-protocol analysis, vaccine efficacy was assessed by analyzing cases starting 14 days after the second dose of vaccination, and (**b**) in the modified intention-to-treat population, cumulative incidence curves are given starting after randomization.

A total of 175 COVID-19 cases were observed among 915 participants from the randomization date to the data cut-off date, which was a median of 132 (IQR 89–133) days of follow-up with an incidence rate of 652.35 per 1000 person-years (95% CI 592.7–709.9). A total of 96 (13.11%) symptomatic COVID-19 cases were observed in the mPP group over 90 days (IQR 86–90) of follow-up (incidence rate 597.15 per 1000 person-years, 95% CI 516.2–672.8), of whom 61 (16.44%) were among CoronaVac recipients (n = 371), with an incidence rate of 762.68 per 1000 person-years (95% CI 654.2–850.5). Among TURKOVAC recipients (n = 361), 35 (9.70%) contracted COVID-19, with an incidence rate of 433.26 per 1000 person-years (95% CI 322.4–546.9). The relative risk reduction with TURKOVAC compared to CoronaVac was 41.03% (95% CI 12.95–60.06) for preventing PCR-confirmed symptomatic COVID-19 with an absolute risk reduction of 6.75% (95% CI 1.86–11.63). Cumulative incidences of COVID-19 events in the TURKOVAC and CoronaVac arms are given in Figure 2. As the study products were inactivated vaccines, a single dose was not expected to be as efficacious as two doses; hence, the primary efficacy analysis was done in the mPP group. There was only one case of hospitalization for symptomatic COVID-19 in the mPP analysis set, and that was in the CoronaVac arm. There was no severe case with regard to the WHO Clinical Progression scale in the cohort during the follow-up period (Table S5).

### 3.3. Immunogenicity

**Figure 3:**
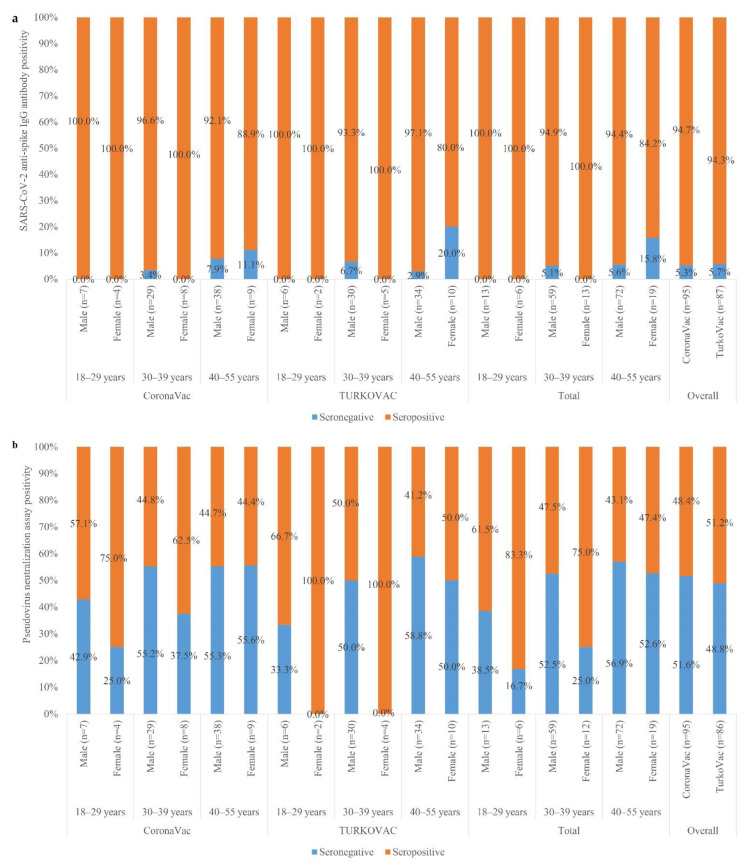
Seropositivity rates in the CoronaVac and TURKOVAC arms 14 days later than the second dose and its distribution according to age and sex: (**a**) for SARS-CoV-2 anti-spike IgG antibodies, (**b**) for pseudovirus neutralization assay. SARS-CoV-2, severe acute respiratory syndrome coronavirus-2; IgG, immunoglobulin G.

Follow-up samples for immunogenicity studies were collected within a median of 33.5 (Q1–Q3: 21–55) days after the second dose of the vaccine. The seroconversion rates for anti-SARS-CoV-2 spike antibodies were 94.7% among CoronaVac (n = 95) and 94.3% among TURKOVAC (n = 87) recipients (*p* = 1.000), who could be sampled for immunogenicity studies (Figure 3a). The pseudovirus neutralization assay yielded positivity rates of 48.4% among CoronaVac (n = 95) and 51.2% among TURKOVAC (n = 86) recipients (*p* = 0.713) (Figure 3b).

### 3.4. Safety

**Figure 4:**
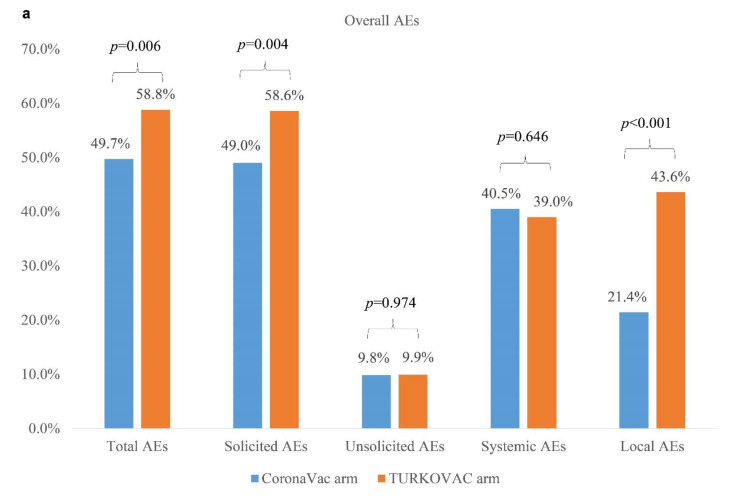

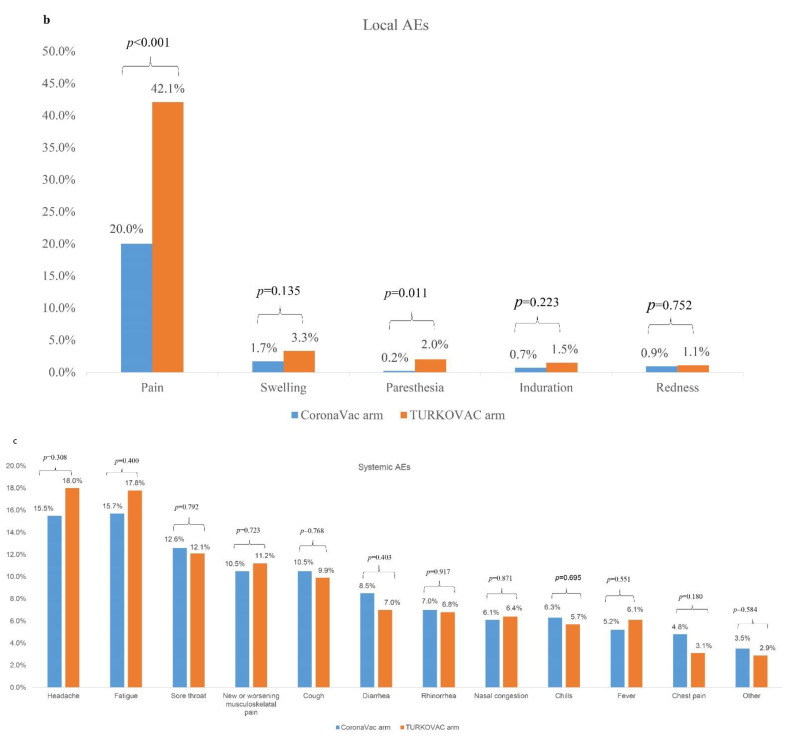
(**a**) Overall adverse events, (**b**) local adverse events, and (**c**) systemic adverse events in the CoronaVac and TURKOVAC arms. AEs, adverse events.

Analyses of AEs were performed in the mITT group (n = 915) (Figure 1). Both vaccines exhibited a satisfactory safety profile without any grade four AEs or fatalities during the study period. A total of 1650 AEs were reported for 496 subjects, which resolved within a median of two (IQR 1–4) days. Overall, AEs were reported by 268 (58.8%) participants in the TURKOVAC group and 228 (49.7%) participants in the CoronaVac group (*p* = 0.006) (Figure 4a). Solicited AEs were higher in the TURKOVAC arm (n = 267, 58.6%) compared with the CoronaVac arm (n = 225, 49.0%) (*p* = 0.004). Unsolicited AEs had a relatively low incidence in both arms (Figure 4a). A comprehensive breakdown of AEs is given in the Supplementary Material S6 (Table S6).

Local reactions were more commonly reported among the TURKOVAC recipients (n = 199, 43.6%) than the CoronaVac recipients (n = 98, 21.4%) (*p* < 0.001). The most common solicited local reaction was inoculation site pain which occurred significantly more frequently with TURKOVAC (n = 192, 42.1%) compared with CoronaVac (n = 92, 20%) (*p* < 0.001). Other local AEs, including swelling, paresthesia, and induration, were rare and not significantly different in both arms except for paresthesia which was higher with TURKOVAC (n = 9, 2.0%) compared with CoronaVac (n = 1, 0.2%) (*p* = 0.011) (Figure 4b).

Systemic AEs were infrequent in both arms without any significant difference between the two vaccines. The most commonly reported systemic AEs were headache and fatigue (n = 153, 16.7% for both) (Figure 4c).

A total of 7 (0.8%) serious AEs were reported during the study period; 3 (0.7%) were in the TURKOVAC arm, and 4 (0.9%) were in the CoronaVac arm (Supplementary Material S6; Table S7). One participant in the CoronaVac arm and three participants in the TURKOVAC arm were hospitalized for symptomatic COVID-19; however, only one participant in the CoronaVac arm was in the period beyond 14 days after the second dose. The distribution of COVID-19 cases with regard to the WHO Clinical Progression Scale is given in the Supplementary Material S5, Table S5.

## 4. Discussion

This interim analysis demonstrated that TURKOVAC was safe and non-inferior to CoronaVac to prevent symptomatic COVID-19 after 14 days of the second dose with a relative risk reduction of 41.03% (95% CI 12.95–60.06) among people 18–55 years old. There was only one patient requiring hospitalization for COVID-19 beyond 14 days after the second dose of the trial vaccine, who was in the CoronaVac arm. As this was a non-inferiority trial, we cannot comment on the individual efficacy of each vaccine [15].

There were certain reasons for the choice of another inactivated vaccine as the active comparator rather than an mRNA vaccine. First of all, the volunteers screened for this phase III trial were mainly individuals who did not get the authorized vaccines in Türkiye for many months after the COVID-19 vaccine roll-out started, and several of them might have had hesitancy to receive the mRNA vaccine. Hence, we believe that having an inactivated vaccine as a comparator definitely increased the chances of volunteer recruitment. Secondly, inactivated vaccines have shown lower efficacy against symptomatic COVID-19 compared to mRNA vaccines [16]. When a non-inferiority trial utilizes a vaccine that has an efficacy ≥ 90%, the power of that study is low to confirm the efficacy of a worthwhile vaccine that is safe, efficacious, and fulfills the WHO criteria for authorization, having a favorable 60–70% level of efficacy [17].

The majority of the volunteers in this study were enrolled during the period when the Delta variant was the dominant VOC in Türkiye. Fiolet and colleagues [18] recently published a comprehensive analysis of COVID-19 vaccines and demonstrated that all vaccines appear to be safe and efficacious in preventing severe COVID-19, hospitalization, and death against the VOC (before the emergence of the Omicron). The efficacy of CoronaVac in preventing symptomatic COVID-19 and COVID-19-related hospitalization after 14 days of the second dose was 83.5% and 100%, respectively [11]. Real-world studies also prove that although inactivated vaccines offer limited protection against symptomatic disease, their effectiveness against hospitalization, severe disease, and mortality is quite high after full-dose immunization, even in the face of the Delta variant [18]. A prospective national cohort study in Chile using CoronaVac reported 87.5% effectiveness in preventing hospitalization, 90.3% effectiveness in preventing intensive care unit (ICU) admission, and 86.3% effectiveness in preventing COVID-19-related death [19]. Real-world data from Malaysia with a different methodology demonstrated effectiveness estimates of 72.0% in preventing ICU admission and 82.4% in preventing deaths for full-dose CoronaVac vaccination [20]. Cerqueira-Silva et al. [21] showed that the effectiveness of CoronaVac against death was 84.8% (95%CI 77.1–89.9) in those <60 years.

The seroconversion rate after two doses of TURKOVAC was 94.4%, and 52.2% of samples yielded neutralizing activity in the pseudovirus neutralization array. Seropositivity rates of neutralizing antibodies in a Chilean population immunized with CoronaVac were reported as over 80% for the Alpha and Gamma variants, over 75% for the Delta variant, and over 60% for the Beta variants [22]. With the emergence of Omicron, even the mRNA vaccines shown to have the highest efficacy lost protection. Two Omicron variants (HKU691 and HKU344-R346K) were tested for the neutralization capacity of BNT162b2 and CoronaVac recipients. Only about 20% of BNT162b2 recipients and none of the CoronaVac recipients had detectable neutralizing antibody levels against either Omicron isolate [23].

We utilized an mPP analysis set that included a follow-up period of 90 days (beyond 14 days after the second dose of the trial vaccine). There are two main reasons for that: (1) The MoH of Türkiye issued a regulation that those who have received two doses of CoronaVac should receive a booster dose three months after the second dose, (2) immunity with inactivated vaccines wanes rapidly over time, especially beyond three months and very low neutralizing antibody concentrations were detected at six months after two doses of CoronaVac [24,25]. The waning immunity was well demonstrated by Zeng et al. [26] as the initial neutralizing antibody response from two doses of CoronaVac declined to near or below the lower limit of seropositivity after six months. However, a third dose of CoronaVac given at a longer interval (eight months) after the second dose boosted the immunity, corresponding to nearly three-fold to five-fold increases in neutralizing antibody titers compared to titers 28 days after the second dose, indicating a good immune memory. Hence, it is clear that given the immunological properties of inactivated vaccines and the data from the efficacy and effectiveness studies, a third dose of CoronaVac- and TURKOVAC- can be integrated into the primary vaccination scheme or as a homologous vaccine boost to provide longer lasting immunity. Moreover, Bartsch et al. [27] demonstrated that vaccine-induced spike-specific antibodies continue to recognize the Omicron variant virus and recruit Fc-receptors. Induction of cellular responses against other SARS-CoV-2 proteins by CoronaVac may confer an advantage compared to other vaccines utilizing the spike protein of the Wuhan strain.

The tolerability profile of the two vaccines was very good. Overall, both vaccines had a very good safety profile, yet TURKOVAC has caused more frequent overall, solicited, and local AEs than CoronaVac. Injection site pain was the most common AE driving the difference. While CoronaVac has 0.225 mg of aluminum hydroxide per 0.5 mL dose, TURKOVAC has 0.5 mg of aluminum hydroxide per 0.5 mL dose, which might be responsible for the significantly more frequent local AEs in the TURKOVAC arm. This adjuvant dose is within the allowed limits as WHO allows up to 1.25 mg of aluminum hydroxide per dose of vaccine [28]. While the majority of the AEs were grade one, there were three patients hospitalized for COVID-19 in the TURKOVAC arm, two of which were diagnosed before 14 days had passed after the second vaccination, and one was diagnosed after the first vaccination. None of them required oxygen therapy during hospitalization, and all of them were discharged in good health.

Designing phase III trials, utilizing a placebo arm, is not possible for ethical reasons; pursuing a non-inferiority trial brought into its own challenges when some of the screened volunteers did not want to accept the active comparator vaccine, CoronaVac. Recruiting volunteers to studies where there is a high coverage for community vaccination and planning for sample size and dosing schedule, given the ever-changing milieu of the pandemic with the newly emerging VOC, are real challenges. We did not have the chance to amend the protocol to plan for a new sample size with regard to the rapidly increasing COVID-19 incidence rate in the community. Additionally, during the course of the present study, the Delta variant was the most frequent VOC in Türkiye, while the phase III trial of CoronaVac was run before the emergence of the Delta variant.

Nevertheless, this interim analysis evaluated a rather long period of follow-up data, which was a median of 132 days from randomization when compared to 43 days in the phase III trial of CoronaVac [11]. However, the study has several limitations. First of all, the study was closed to recruitment far before reaching the targeted sample size as the primary outcome was attained, TURKOVAC was granted EUA, and the vaccine was integrated as an option for community vaccine roll-out. Moreover, there was a significant proportion of volunteers who turned out to be seropositive for SARS-CoV-2 antibody and dropped out from the mPP analysis set. Secondly, the study population consisted of relatively young, healthy, and socially active individuals with a low prevalence of chronic diseases but a high risk of contracting COVID-19. Hence, the generalizability of the findings to the whole population is not possible. There were very few hospitalized COVID-19 cases, limiting our ability to make generalized conclusions about severe disease. Although this was a non-inferiority study, the comparison of the results of this trial with those of the phase III trial of CoronaVac is not possible as that trial utilized a 0–14 day dosing schedule at a time when the VOC was not widely circulating. The study did not have an objective to evaluate the efficacy of a two-dose regimen of TURKOVAC and two-dose regimen of CoronaVac for the prevention of symptomatic COVID-19, or for the hospitalization and death among RT-PCR-confirmed severe COVID-19 cases with regards to different VOC. Further analyses are required to evaluate the relevance of TURKOVAC on emerging VOC. We can only report preliminary immunogenicity data as the analyses of the sequential serum-neutralizing antibody titers, and T-cell responses are still ongoing.

## 5. Conclusions

Our results demonstrated that TURKOVAC is at least as efficacious as CoronaVac and has a very good safety profile in a population between 18–55 years of age. As this is an interim analysis that included a low number of volunteers, further data are still needed on the performance of TURKOVAC to demonstrate the efficacy of the vaccine against different VOC and the duration of protection as well as to assess the safety and efficacy in the elderly population, adolescents, younger children, and individuals with certain chronic diseases. Given that inactivated vaccines can be shipped and shelved for over three years at regular fridge temperatures of 2–8 °C, they are granted a clear superiority to be used in low-and middle-income countries. Hence, TURKOVAC is as safe and efficacious a vaccine as CoronaVac and holds promise to increase the availability of COVID-19 vaccines, especially in resource-scarce settings across the world, to prioritize the health benefits both for the individual and the public. 

## 6. Patents

Aykut Ozdarendeli is the named inventor on patent applications covering inactivated COVID-19 vaccine development.

## Figures and Tables

**Table 1:** Main characteristics of the study population.

Characteristics *	CoronaVac Arm n = 459	TURKOVAC Arm n = 456
Age, years, Median (IQR)	39 (32–44)	38 (34–44)
Age groups, n (%)		
18–29 years	78 (17)	69 (15.1)
30–39 years	170 (37)	188 (41.2)
40–55 years	211 (46)	199 (43.6)
Sex, n (%)		
Male	325 (70.8)	340 (74.6)
Female	134 (29.2)	116 (25.4)
BMI, kg/m^2^, Median (IQR)	25.96 (23.53–29.04)	26.26 (23.56–28.60)
BMI, kg/m^2^, n (%)		
<25	176 (38.3)	172 (37.7)
≥30	85 (18.5)	69 (15.1)
25–30	198 (43.1)	215 (47.1)
Medical History, n (%)		
Hypertension	6 (1.3)	5 (1.1)
Diabetes mellitus	2 (0.4)	4 (0.9)
Allergic conditions	5 (1.1)	12 (2.6)
Neurological and psychiatric status	6 (1.3)	6 (1.3)
Respiratory diseases	5 (1.1)	1 (0.2)
Thyroid diseases	7 (1.5)	1 (0.2)
Presence of any medical history	69 (15)	74 (16.2)
Concomitant drug use **	49 (11.8)	49 (12.0)

IQR, interquartile range; BMI, body mass index. * All participants were Caucasians for both vaccine arms. ** The analysis for concomitant drug use was conducted with over 823 participants due to missing data.

## Data Availability

Proposals should be submitted to the sponsor, Health Institutes of Türkiye, who holds all the financial and intellectual rights of the study and the study vaccine TURKOVAC.

## Supplementary Materials

The following supporting information can be downloaded at: https://www.mdpi.com/article/10.3390/vaccines10111865/s1, Supplementary Material S1: Procedures on the screening/Day 1 and Day 28 visits, unscheduled visits and the composition and pharmacological properties of the investigational product; Supplementary Material S2: COVID-19 Surveillance; Supplementary Material S3: Safety monitoring; Supplementary Material S4: List of participating sites; Supplementary Material S5: Distribution of COVID-19 cases with regard to the WHO Clinical Progression Scale, Table S5: COVID-19 severity and symptoms of the cases in the efficacy analysis with regard to WHO Clinical Progression Scale; Supplementary Material S6: Adverse events, Table S6: Distribution of adverse events; Supplementary Material S7: The TURKOVAC Study Group, Table S7: Distribution of serious adverse events.

## References

[1] Jindal H.A., Sahoo S.S., Jamir L., Kedar A., Sharma S., Bhatt B. (2021). Higher coronavirus disease-19 mortality linked to comorbidities: A comparison between low-middle income and high-income countries. J. Educ. Health Promot..

[2] Brüssow H., Zuber S. (2021). Can a combination of vaccination and face mask wearing contain the COVID-19 pandemic?. Microb. Biotechnol..

[3] Ritchie H., Mathieu E., Rodés-Guirao L., Appel C., Charlie Giattino C., Ortiz-Ospina E., Hasell J., Macdonald B., Dattani S., Roser M. Coronavirus Pandemic (COVID-19). https://ourworldindata.org/coronavirus.

[4] World Health Organization From Vaccines to Vaccinations: Seventh Meeting of the Multilateral Leaders Task Force on COVID-19 Vaccines, Therapeutics and Diagnostics. https://www.who.int/news/item/22-12-2021-from-vaccines-to-vaccinations-seventh-meeting-of-the-multilateral-leaders-task-force-on-COVID-19-vaccines-therapeutics-and-diagnostics.

[5] McIntyre P.B., Aggarwal R., Jani I., Jawad J., Kochhar S., MacDonald N., Madhi S.A., Mohsni E., Mulholland K., Neuzil K.M. (2022). COVID-19 vaccine strategies must focus on severe disease and global equity. Lancet.

[6] World Health Organization COVID-19 Vaccine Tracker and Landscape. https://www.who.int/publications/m/item/draft-landscape-of-COVID-19-candidate-vaccines.

[7] Li Y.D., Chi W.Y., Su J.H., Ferrall L., Hung C.F., Wu T.C. (2020). Coronavirus vaccine development: From SARS and MERS to COVID-19. J. Biomed. Sci..

[8] Pérez-Then E., Lucas C., Monteiro V.S., Miric M., Brache V., Cochon L., Vogels C.B.F., Malik A.A., De la Cruz E., Jorge A. (2022). Neutralizing antibodies against the SARS-CoV-2 Delta and Omicron variants following heterologous CoronaVac plus BNT162b2 booster vaccination. Nat. Med..

[9] World Health Organization COVID-19 Vaccines WHO EUL Issued. https://extranet.who.int/pqweb/vaccines/vaccinescovid-19-vaccine-eul-issued.

[10] Mallapaty S. (2021). China’s COVID vaccines have been crucial—Now immunity is waning. Nature.

[11] Tanriover M.D., Doğanay H.L., Akova M., Güner H.R., Azap A., Akhan S., Köse Ş., Erdinç F.Ş., Akalın E.H., Tabak Ö.F. (2021). Efficacy and safety of an inactivated whole-virion SARS-CoV-2 vaccine (CoronaVac): Interim results of a double-blind, randomised, placebo-controlled, phase 3 trial in Türkiye. Lancet.

[12] Pavel S., Yetiskin H., Uygut M.A., Aslan A.F., Aydın G., İnan Ö., Kaplan B., Ozdarendeli A. (2021). Development of an inactivated vaccine against SARS-CoV-2. Vaccines.

[13] Ozdarendeli A., Sezer Z., Pavel S.T.I., Inal A., Yetiskin H., Kaplan B., Uygut M.A., Bayram A., Mazicioglu M., Kalin Unuvar G. (2022). Safety and immunogenicity of an inactivated whole virion SARS-CoV-2 vaccine, TURKOVAC, in healthy adults: Interim results from randomised, double-blind, placebo-controlled phase 1 and 2 trials. Vaccine.

[14] WHO Working Group on the Clinical Characterisation and Management of COVID-19 infection (2020). A minimal common outcome measure set for COVID-19 clinical research. Lancet Infect. Dis..

[15] World Health Organization COVID-19 Vaccine Trial Designs in the Context of Authorized COVID-19 Vaccines and Expanding Global Access: Ethical Considerations. https://www.who.int/publications/i/item/WHO-2019-nCoV-Policy-brief-Vaccine-trial-design-2021.1.

[16] Huang Z., Su Y., Zhang T., Xia N. (2022). A review of the safety and efficacy of current COVID-19 vaccines. Front. Med..

[17] Fleming T.R., Krause P.R., Nason M., Longini I.M., Henao-Restrepo A.M. (2021). COVID-19 vaccine trials: The use of active controls and non-inferiority studies. Clin. Trials.

[18] Fiolet T., Kherabi Y., MacDonald C.J., Ghosn J., Peiffer-Smadja N. (2022). Comparing COVID-19 vaccines for their characteristics, efficacy and effectiveness against SARS-CoV-2 and variants of concern: A narrative review. Clin. Microbiol. Infect..

[19] Jara A., Undurraga E.A., González C., Paredes F., Fontecilla T., Jara G., Pizarro A., Acevedo J., Leo K., Leon F. (2021). Effectiveness of an inactivated SARS-CoV-2 vaccine in Chile. N. Engl. J. Med..

[20] Suah J.L., Tok P., Ong S.M., Husin M., Tng B.H., Sivasampu S., Thevananthan T., Appannan M.R., Muhamad Zin F., Mohd Zin S. (2021). PICK-ing Malaysia’s epidemic apart: Effectiveness of a diverse COVID-19 vaccine portfolio. Vaccines.

[21] Cerqueira-Silva T., Oliveira V.A., Boaventura V.S., Pescarini J.M., Júnior J.B., Machado T.M., Flores-Ortiz R., Penna G.O., Ichihara M.Y., de Barros J.V. (2022). Influence of age on the effectiveness and duration of protection of Vaxzevria and CoronaVac vaccines: A population-based study. Lancet Reg. Health Am..

[22] Melo-González F., Soto J.A., González L.A., Fernández J., Duarte L.F., Schultz B.M., Gálvez N.M.S., Pacheco G.A., Ríos M., Vázquez Y. (2021). Recognition of variants of concern by antibodies and T cells induced by a SARS-CoV-2 inactivated vaccine. Front. Immunol..

[23] Lu L., Mok B.W., Chen L.L., Chan J.M., Tsang O.T., Lam B.H., Chuang V.W., Chu A.W., Chan W.M., Ip J.D. (2022). Neutralization of SARS-CoV-2 Omicron variant by sera from BNT162b2 or Coronavac vaccine recipients. Clin. Infect. Dis..

[24] Ju B., Zhou B., Song S., Fan Q., Ge X., Wang H., Cheng L., Guo H., Shu D., Liu L. (2022). Potent antibody immunity to SARS-CoV-2 variants elicited by a third dose of inactivated vaccine. Clin. Transl. Med..

[25] Costa Clemens S.A., Weckx L., Clemens R., Almeida Mendes A.V., Ramos Souza A., Silveira M.B.V., da Guarda S.N.F., de Nobrega M.M., de Moraes Pinto M.I., Gonzalez I.G.S. (2022). Heterologous versus homologous COVID-19 booster vaccination in previous recipients of two doses of CoronaVac COVID-19 vaccine in Brazil (RHH-001): A phase 4, non-inferiority, single blind, randomised study. Lancet.

[26] Zeng G., Wu Q., Pan H., Li M., Yang J., Wang L., Wu Z., Jiang D., Deng X., Chu K. (2022). Immunogenicity and safety of a third dose of CoronaVac, and immune persistence of a two-dose schedule, in healthy adults: Interim results from two single-centre, double-blind, randomised, placebo-controlled phase 2 clinical trials. Lancet Infect. Dis..

[27] Bartsch Y., Tong X., Kang J., José Avendaño M., Serrano E.F., García-Salum T., Pardo-Roa C., Riquelme A., Medina R.A., Alter G. (2021). Preserved Omicron Spike specific antibody binding and Fc-recognition across COVID-19 vaccine platforms. Medrxiv.

[28] World Health Organization (2014). WHO Technical Report Series No. 980. https://www.who.int/biologicals/WHO_TRS_980_WEB.pdf2014.

