# Supplementary material for: Efficacy, Immunogenicity, and Safety of the Two-Dose Schedules of TURKOVAC versus CoronaVac in Healthy Subjects: A Randomized, Observer-Blinded, Non-Inferiority Phase III Trial
Source: Vaccines (Basel). 2022 Nov 4;10(11):1865. doi: 10.3390/vaccines10111865 (PMC9698857; doi:10.3390/vaccines10111865)
Supplement: Supplementary file 1 [file vaccines-10-01865-s001.zip › Supplementary Material S1.pdf]

Supplementary Material S1. Procedures on the screening/Day 1 and Day 28 visits, unscheduled visits and the composition and pharmacological properties of the investigational product

1.1. Procedures

Visit 1: Informed consent was obtained, eligible participants were identified by inclusion/exclusion criteria, medical history and concomitant medications were recorded, physical examination was performed, urine pregnancy test was done for women of childbearing potential, vital signs (body temperature, blood pressure, heart rate, respiratory rate), height, and weight were measured and demographic data were collected in Visit 1. After randomization, first dose of 3 µg/0.5 mL TURKOVAC or 3 µg/0.5 mL CoronaVac vaccine were administered.

Visit 2: Changes in concomitant medications were recorded, urine pregnancy test was repeated for women of childbearing potential, physical examination was performed, vital signs, height, and weight were measured and the second dose of 3 µg/0.5 mL TURKOVAC vaccine or 3 µg/0.5 mL CoronaVac vaccine was administered in Visit 2- Day 28.

Unscheduled visits: Unscheduled visits were issued for all subjects if symptoms occurred. Nasopharyngeal swab was collected for severe acute respiratory syndrome coronavirus-2 (SARS-CoV-2) reverse transcription-polymerase chain reaction (RT-PCR). At these visits, medical history, concomitant medications changes, safety information and Coronavirus disease 2019 (COVID-19) symptoms of subjects were collected.

All subjects were observed for 30 minutes after each vaccination and were advised to record daily any untoward medical events up to 12 months after the last dose. Interactive Web-based Response System (IWRS) called all subjects by phone every day until seventh day after each

vaccination and weekly after one week of the vaccination, to remind them to report any adverse event to research team in their sites or call center. A referral system was established and all serious adverse events (SAEs) were managed and necessary hospitalization/treatment were ensured according to the local regulations.

Symptom-based active surveillance was performed to detect participants with symptoms suggesting COVID-19 during the follow-up. Anyone with at least one of the following symptoms for two days or more underwent PCR testing: Fever or chills; cough; shortness of breath or difficulty in breathing; fatigue; muscle or body pain; headache; loss of smell or new taste; sore throat; nasal congestion or runny nose; nausea or vomiting; and diarrhea. All cases of SARS-CoV-2 infection were classified according to the scale of clinical progression proposed by WHO (*WHO Working Group on the Clinical Characterisation and Management of COVID-19 infection. A minimal common outcome measure set for COVID-19 clinical research. Lancet Infect Dis 2020;20:e192–e197. https://doi.org/10.1016/S1473-3099(20)30483-7.*). The evaluation of hospitalized cases (score 4 or higher) were done daily until the resolution of symptoms. In non-hospitalized cases, the maximum score and duration of symptoms were recorded (score 1-3).

Table S1. Scale of clinical progression of SARS-CoV-2 infection (*WHO Working Group on the Clinical Characterisation and Management of COVID-19 infection. A minimal common outcome measure set for COVID-19 clinical research. Lancet Infect Dis 2020;20:e192–e197. https://doi.org/10.1016/S1473-3099(20)30483-7***)**

Score Description	
Uninfected, viral RNA not detected	0
Asymptomatic, viral RNA detected	1

Symptomatic, independent	2
Symptomatic, need help	3
Hospitalized *, without oxygen	4
Hospitalized, supplemental oxygen by mask or nasal cannula	5
Hospitalized, oxygen by non-invasive or high flow ventilation	6
Intubation and mechanical ventilation, P02 / FiO2 ≥150 or SpO2 / FiO2 ≥ 200	7
Mechanical ventilation P02 / FiO2 <150 (SpO2 / FiO2 <200) or vasopressors	8
Mechanical ventilation P02 / FiO2 <150 (SpO2 / FiO2 <200) and vasopressors, dialysis or extracorporeal oxygenation	9
Death	10

** If hospitalization is for isolation only, record the status with an outpatient*

1.2. Study products

CoronaVac is prepared by a novel coronavirus (CZ02 Strain) inoculated in African green monkey kidney cells (Vero Cells). It is a milky white suspension liquid, which can be layered due to precipitation and quickly dispersed. One dose of COVID-19 vaccine contains 600 SU (1 µg of antigen equals to 200 SU) of SARS-CoV-2 antigen in a 0.5 mL aqueous suspension for injection with 0.45 mg/mL of aluminium. The only active substance in the COVID-19 Vaccine is the inactivated SARS-CoV-2 whole-virion as SARS-CoV-2 antigen developed and manufactured by Sinovac Life Sciences, China. The Medicinal Product is developed entirely based on the features of this active substance; thus, there is no compatibility concern for using this drug substance in the formulation. The components in CoronaVac are shown below:

Composition of CoronaVac:

Name of the ingredient	Content per dose
SARS-CoV-2 antigen	600 SU
Aluminium hydroxide (calculated as per aluminium)	0.45 mg/mL
Sodium chloride	9 mg/mL
Phosphate buffer solution*	5.0 mmol/L
Sodium hydroxide	q.s.
Water for injection	q.s. to 0.5 mL

q.s., Quantum satis

*The phosphate buffer is prepared by monosodium hydrogen phosphate and disodium hydrogen phosphate; the content is calculated according to the phosphate ion concentration in the vaccine.

The TURKOVAC study vaccine includes inactivated COVID-19 virus. The dosage of TURKOVAC for this study was 3 µg/0.5 mL. Study vaccine is a white suspension containing SARS-CoV-2 inactivated antigen and ALUM GEL (AlOH-INVIVOGEN) as an adjuvant. It has been formulated as a final concentration of 0.05% (0.5 mg/dose) from its 2% suspension. It does not contain preservative or stabilizer. Sterility test was performed according to Pharmacopeia method and final product and in-process controls were found to be sterile. ALUM GEL adjuvant (2% initial concentration), is adsorbed by pre-calculating the formulation volume according to the desired viral antigen ratio in the final product and mixing it with the antigen buffered in

Phosphate Buffer Saline at 25°C at pH 7.2 - 7.4. Meanwhile, Phosphate Buffer Saline is added to the final volume. Aluminum content in the final volume should be 0.05% - 0.06%. The Lowry protein analysis method was used in the experiments, and the protein ratios between before and after adsorption were compared and the adsorption rate was found to be over 98.5%. The reason for selecting ALUM GEL as adjuvant is that its adsorption rate has higher binding capacity than to other aluminum-based adjuvants and it stimulates immunity at a higher level depending on the antigen release rate in in-vivo trials. The components in TURKOVAC are shown below:

**Composition of the finished product**	**QUANTITY**
SARS-CoV-2 Antigen	3 µg/0.5 mL
Alum Gel	0.5 mg/0.5 mL
Phosphate buffer solution*	K.M 0.5 mL

The recommended site of administration for the study vaccines was left deltoid of the upper arm by intramuscular injection. A 2-mL syringe with a 22 Gauge needle was used for administration of vaccines.

1.3.Randomization and masking

Unblinded nurses were assigned to prepare the study vaccines for injection. They had no study functions other than study vaccine management, documentation, accountability, preparation, and administration. They were not involved in participant evaluations and did not reveal the identity of study vaccine to either the participant or the blinded study site personnel involved in the conduct of the study. Each enrolled participant was assigned a code and the unblinded nurse stuck the coded labels on the syringes and prepared labeled syringes filled with

the study vaccine in a separate room to make sure that the contents of the syringes were not visible. Access to the randomization code was strictly controlled by the nurse in the room where the study vaccines were located. All other site staff performing study-related assessments remained blinded for the duration of the study. The Sponsor also remained blinded to individual participant vaccine assignments until the time of study unblinding at the analysis of the primary end point.
