# Supplementary material for: Efficacy, Immunogenicity, and Safety of the Two-Dose Schedules of TURKOVAC versus CoronaVac in Healthy Subjects: A Randomized, Observer-Blinded, Non-Inferiority Phase III Trial
Source: Vaccines (Basel). 2022 Nov 4;10(11):1865. doi: 10.3390/vaccines10111865 (PMC9698857; doi:10.3390/vaccines10111865)
Supplement: Supplementary file 1 [file vaccines-10-01865-s001.zip › Supplementary Material S2.pdf]

Supplementary Material S2. COVID-19 Surveillance

2.1. IVRS (Interactive Voice Response System) script for 3 days:

"Hi,

Welcome to the COVID-19 local vaccine study, symptom tracking phoneline.

Now, we kindly request you to answer the questions about the side effects occurred in the vaccination site.

If there is pain, redness, stiffness or swelling in the vaccination site, please dial **1;** if there is not, please dial **0**."

"If there is pain in the vaccination site, dial 1; if not, dial 0."

If 1 is dialed: "If your pain is severe enough to require you to visit the emergency room or be hospitalized please dial **1**; if it is not, please dial **0**."

If 1 is dialed : "The research team has been notified."

"If there is redness, dial 1; if not, dial 0."

"If the redness in the vaccination site is severe enough to require you to visit the emergency room or be hospitalized, please dial **1**; if it is not, please dial **0**."

If 1 is dialed : "The research team has been notified."

"If there are stiffness and swelling, dial 1; if there is not, dial 0."

If 1 is dialed : "If the stiffness or swelling in the vaccination site is severe enough to require you to visit the emergency room or be hospitalized, please dial **1**; if it is not, please dial **0**."

If 1 is dialed : "The research team has been notified."

"If you have any of the symptoms that we are going to list now in groups, dial **1**; if you do not, dial **0**."

"If there is chest pain, shortness of breath or difficulty in breathing, dial **1;** if there is not, dial **0**."

"If you have chest pain dial **1;** if you do not, dial **0**.

If 1 is dialed : "If your chest pain is severe enough to require you to visit the emergency room or be hospitalized, please dial **1;** if it is not, please dial **0**. "

If 1 is dialed : "The research team has been notified."

If 0 is dialed : "If you have shortness of breath dial **1;** if you do not, dial **0**."

If 1 is dialed : "If your shortness of breath is severe enough to require you to visit the emergency room or be hospitalized, please dial **1**; if it is not, please dial **0**."

If 1 is dialed : "The research team has been notified**."**

If 0 is dialed : "If you have difficulty in breathing, dial **1**; if you do not, dial **0**."

If 1 is dialed : "If your difficulty in breathing is severe enough to require you to visit the emergency room or be hospitalized, please dial **1**; if it is not, please dial **0**.

If 1 is dialed : "The research team has been notified."

If 0 is dialed : " If you have fever, dial **1**; if you do not, dial **0**."

If 1 is dialed : **“**If Your body temperature is 38°C or above 38°C, please dial **1**; if it is below 38°C , please dial **0**.”

If 1 is dialed : **“**The research team has been notified**.”**

If 0 is dialed : **”**If you have cough, dial **1**; if you do not, dial **0**.”

If 1 is dialed : **“**If your cough is severe enough to require you to visit the emergency room or be hospitalized, please dial **1**; if it is not, please dial **0**. **“**

If 1 is dialed : **“**The research team has been notified.”

If 0 is dialed : **”**If there is tremble, chill or nasal congestion, dial **1**; if there is not dial **0**.”

“If you have tremble, dial **1**; if you do not, dial **0**.”

If 1 is dialed : **“**If your tremble is severe enough to require you to visit the emergency room or be hospitalized, please dial **1**; if it is not, please dial **0**.”

If 1 is dialed: **“**The research team has been notified.”

If 0 is dialed : **“**If you have chill, dial **1**; if you do not, dial **0.”**

If 1 is dialed : **“**If your chill is severe enough to require you to visit the emergency room or be hospitalized, please dial **1**; if it is not, please dial **0.”**

If 1 is dialed : **“**The research team has been notified.”

If 0 is dialed : “If you have nasal congestion, dial **1**; if you do not, dial **0**.”

If 1 is dialed : “If your nasal congestion is severe enough to require you to visit the emergency room or be hospitalized, please dial **1**; if it is not, please dial **0**.”

If 1 is dialed : “The research team has been notified.”

If 0 is dialed : “If there is sore throat, muscle pain, headache or tiredness, dial **1**; if there is not, dial **0.”**

-“ If you have sore throat, dial **1;** if you do not, dial **0**.”

If 1 is dialed : “If your sore throat is severe enough to require you to visit the emergency room or be hospitalized, please dial **1**; if it is not, please dial **0**.”

If 1 is dialed : “The research team has been notified.”

If 0 is dialed :”If you have muscle pain dial **1**; if you do not, dial **0**.”

If 1 is dialed : “If the muscle pain is in the vaccination site, dial **1**; if it is in another site, please dial **0**.”

If 1 and/or 0 is dialed : “If your muscle pain is severe enough to require you to visit the emergency room or be hospitalized, please dial **1**; if it is not, please dial **0.”**

If 1 is dialed : “The research team has been notified.”

If 0 is dialed : “If you have headache, dial 1; if you do not, dial 0**.”**

If 1 is dialed : “If your headache is severe enough to require you to visit the emergency room or be hospitalized, please dial **1**; if it is not, please dial **0**.”

If 1 is dialed : “The research team has been notified.”

If 0 is dialed : “ If you have tiredness, dial **1**; if you do not, dial **0**.”

If 1 is dialed : “If your tiredness is severe enough to require you to visit the emergency room or be hospitalized, please dial **1**; if it is not, please dial **0**.”

If 1 is dialed : “The research team has been notified.”

If 0 is dialed : “If there is diarrhea, vomiting, loss of sense of smell or loss of sense of taste, dial **1**; if there is not, dial **0**.”

-“ If you have diarrhea, dial **1**; if you do not, dial **0**.”

If 1 is dialed : “If your diarrhea is severe enough to require you to visit the emergency room or be hospitalized, please dial **1**; if it is not, please dial **0**.”

If 1 is dialed : “The research team has been notified.”

If 0 is dialed : “ If you have vomiting, dial **1**; if you do not, dial **0**.”

If 1 is dialed : “If vomiting is severe enough to require you to visit the emergency room or be hospitalized, please dial **1**; if it is not, please dial **0.”**

If 1 is dialed : “The research team has been notified**.”**

If 0 is dialed : “If there is loss of sense of smell, dial **1**; if there is not, dial **0.”**

If 1 is dialed : “If the loss of your sense of smell is severe enough to require you to visit the emergency room or be hospitalized, please dial **1**; if it is not, please dial **0.”**

If 1 is dialed : “The research team has been notified.”

If 0 is dialed : “If there is loss of sense of taste, dial 1; if there is not, dial 0.”

If 1 is dialed : “If the loss of your sense of taste is severe enough to require you to visit the emergency room or be hospitalized, please dial **1**; if it is not, please dial **0.”**

If 1 is dialed : “The research team has been notified.”

If 0 is dialed : ”Thank you for your answers, we wish you a healthy day**.”**

-Script End-

2.2. IVRS system script for 3 weeks:

“Hi,

Welcome to the COVID-19 vaccine study, symptom tracking phoneline.

If you have any of the symptoms that we are going to list now in groups, please dial 1, if you do not dial 0.”

-Fever

If 1 is dialed: “If Your body temperature is 38°C or above 38°C, please dial **1**; if it is below 38°C, please dial **0**.”

If 1 is dialed : “The research team has been automatically notified about the problem you have.”

If 0 is dialed:

-Cough

If 1 is dialed: “If your cough is severe enough to require you to visit the emergency room or be hospitalized, please dial **1**, if it is not, please dial **0**.”

If 1 is dialed : “The research team has been automatically notified about the problem you have.”

If 0 is dialed :

- Shortness of breath

If 1 is dialed : “If your shortness of breath is severe enough to require you to visit the emergency room or be hospitalized, please dial **1**, if it is not, please dial **0**.”

If 1 is dialed : “The research team has been automatically notified about the problem you have.”

If 0 is dialed:

-Trembling

If 1 is dialed : “If your trembling is severe enough to require you to visit the emergency room or be hospitalized, please dial **1**, if it is not, please dial **0**.”

If 1 is dialed : “The research team has been automatically notified about the problem you have.”

If 0 is dialed :

-Chill

If 1 is dialed: “If your chill is severe enough to require you to visit the emergency room or be hospitalized, please dial **1**, if it is not, please dial **0**.”

If 1 is dialed : “The research team has been automatically notified about the problem you have.”

If 0 is dialed:

-Nasal congestion

If 1 is dialed : “If your nasal congestion is severe enough to require you to visit the emergency room or be hospitalized, please dial **1**, if it is not, please dial **0**.”

If 1 is dialed : “The research team has been automatically notified about the problem you have.”

If 0 is dialed:

-Sore throat

If 1 is dialed: “If your sore throat is severe enough to require you to visit the emergency room or be hospitalized, please dial **1**, if it is not, please dial **0**.”

If 1 is dialed : “The research team has been automatically notified about the problem you have.”

If 0 is dialed:

-Tiredness

If 1 is dialed : “If your tiredness is severe enough to require you to visit the emergency room or be hospitalized, please dial **1**, if it is not, please dial **0**.”

If 1 is dialed : “The research team has been automatically notified about the problem you have.”

If 0 is dialed :

-Muscle pain

If 1 is dialed :”If your muscle pain is in the vaccination site, please dial **1**, if it is in another site, please dial **0**.”

If 1 and/or 0 is dialed: “If your muscle pain is severe enough to require you to visit the emergency room or be hospitalized, please dial **1**, if it is not, please dial **0**.”

If 1 is dialed : “The research team has been automatically notified about the problem you have.”

If 0 is dialed :

-Headache

If 1 is dialed : “If your headache is severe enough to require you to visit the emergency room or be hospitalized, please dial **1**, if it is not, please dial **0**.”

If 1 is dialed : “The research team is automatically notified about the problem you have.”

If 0 is dialed :

-Diarrhea

If 1 is dialed : "If your diarrhea is severe enough to require you to visit the emergency room or be hospitalized, please dial **1**, if it is not, please dial **0**."

If 1 is dialed : "The research team has been automatically notified about the problem you have."

If 0 is dialed :

-Vomiting

If 1 is dialed : "If your vomiting is severe enough to require you to visit the emergency room or be hospitalized, please dial **1**, if it is not, please dial **0**."

If 1 is dialed : "The research team has been automatically notified about the problem you have."

If 0 is dialed :

-Loss of sense of smell

If 1 is dialed : "If the loss of your sense of smell is severe enough to require you to visit the emergency room or be hospitalized, please dial **1**, if it is not, please dial **0**."

If 1 is dialed : "The research team has been automatically notified about the problem you have."

If 0 is dialed :

-Loss of sense of taste

If 1 is dialed : "If the loss of your sense of taste is severe enough to require you to visit the emergency room or be hospitalized, please dial **1**, if it is not, please dial **0**."

If 1 is dialed : "The research team has been automatically notified about the problem you have."

If 0 is dialed :

"Have you been told that you are a COVID 19 Patient since the time we last contacted with you?"

"If Yes, please dial **1**, if No, please dial **0**."

If 1 is dialed : "The research team has been automatically notified about the problem you have."

If 0 is dialed :

"If you have experienced any situation that requires you to visit a doctor or a hospital or if a doctor or a nurse has visited you, please dial **1**, if No, please dial **0**. "

If 1 is dialed : "Please explain the details of that visit after the tone."

"Have you received any prescription or nonprescription drugs or vitamins for the changes in your health?"

"If Yes, please dial **1**, if No please dial **0**."

If 1 is dialed : "Please tell the name of the drug you received after the tone."

“Have you ever had close contact with anybody who was positive for COVID-19 test?“

“If Yes, please dial **1**, if No please dial **0.”**

If 1 is dialed : “The research team has been automatically notified about the problem you have.”

“If you have experienced any problem other than these issues we listed above, please dial **1**, if you have not, please dial **0**.”

If 1 is dialed : “We kindly request you to briefly explain the problem you experienced after the tone.”

If 0 is dialed : “Thank you for your answers, we wish you a healthy day.”

-Script End-

2.3. COVID-19 diagnosis and management

At the COVID-19 testing visit, a nasopharyngeal and pharyngeal swab was obtained for PCR testing. Appropriate medical management was provided to all participants at the respective COVID-19 hospital. After development of a symptom, if the first PCR result was negative, the second PCR test was performed after 24 to 48 hours. If the second test was negative, participants would be followed-up according to the COVID-19 algorithm published by the Ministry of Health. Symptomatic participants would be regularly reviewed over phone calls and home visits and will be managed until the symptoms recover for patients not requiring hospitalization. A detailed case report form would be completed describing the clinical course and outcome for all hospitalized and non-hospitalized COVID-19 patients. If participants were diagnosed with COVID-19, they would be followed-up according to the COVID-19 algorithm published by the Ministry of Health and an adverse event form would be completed.
