# Supplementary material for: Efficacy, Immunogenicity, and Safety of the Two-Dose Schedules of TURKOVAC versus CoronaVac in Healthy Subjects: A Randomized, Observer-Blinded, Non-Inferiority Phase III Trial
Source: Vaccines (Basel). 2022 Nov 4;10(11):1865. doi: 10.3390/vaccines10111865 (PMC9698857; doi:10.3390/vaccines10111865)
Supplement: Supplementary file 1 [file vaccines-10-01865-s001.zip › Supplementary Material S3.pdf]

Supplementary Material S3. Safety monitoring

3.1. Monitoring

Academicians from different institutions were selected for the independent Data and Safety Monitoring Board. In this context, specialists experienced in infectious diseases and clinical microbiology, public health-epidemiology, social pediatrics, cardiology and internal medicine and vaccination/adverse effects were included in the group. Data and Safety Monitoring Board monitored the quality of evidence, adverse event following, revisions in line with the current literature, individual privacy, and data reliability from the planning stage to the end of the study and oversaw if the practices were carried out in accordance with Good Clinical Practice and Human Rights Declaration conditions. In addition to DSMB, Health Institutes of Türkiye as the sponsor of the study, was responsible for ensuring the proper conduct of the study, in accordance with the Declaration of Helsinki (Amended Fortaleza, Brazil, 2013) and Good Clinical Practices (GCP) including, but not limited to, protocol adherence and the validity of the data recorded in the database.

3.2. Reporting Procedures for SAEs

Any SAE was reported to Data and Safety Monitoring Board (DSMB), Ethics Committee, Ministry of Health, sponsor and clinical study monitor within 24 hours of the investigator's first knowledge of the event, regardless of the presumed relationship to the investigational product. Chest pain and/or dyspnea was managed according to the flow chart prepared within the scope of the study. All AEs were assessed by study investigators for severity and causality. Any AE assessed by study investigators as possibly, probably, or definitely related to study product was defined as adverse reaction.

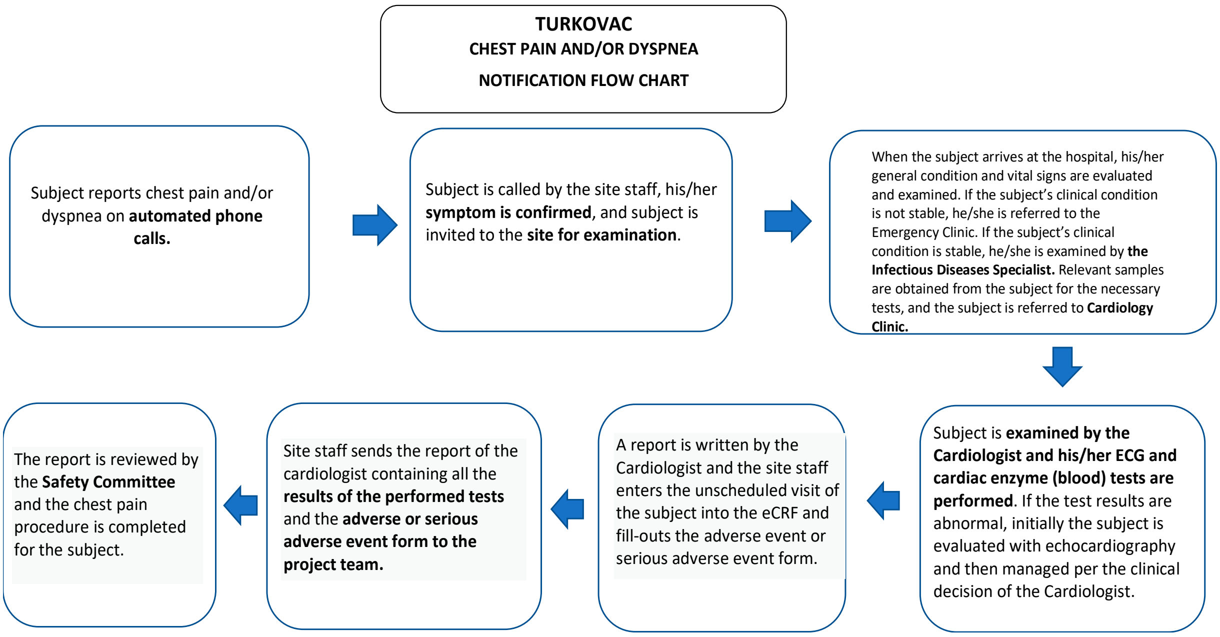

Fig. S1. Management algorithm of chest pain and/or dyspnea

3.3. Assessment of severity of adverse events

The severity of clinical adverse events was assessed according to scales based on Food and Drug Administration (FDA) toxicity grading scales for vaccine clinical trials, as shown in the tables below.

Table S2. Severity grading criteria for local adverse events

	Grade 1	Grade 2	Grade 3	Grade 4
Pain	Not affecting or slightly affecting physical activity	Affecting physical activity	Affecting daily life	Loss of basic self-care ability,

				or hospitalization
Induration*	Diameter 2.5 to <5 cm or area 6.25 to <25 cm^2 without affecting or slightly affecting daily life	5 to <10 cm in diameter or 25 to <100 cm^2 in area or affecting daily life	Diameter ≥10 cm or area ≥100 cm^2 or ulceration or secondary infection or phlebitis or aseptic abscess or wound drainage or seriously affecting daily life	Abscess, exfoliative dermatitis, dermal or deep tissue necrosis
Swelling*	Diameter 2.5 to <5 cm or area 6.25 to <25 cm^2 without affecting or slightly affecting daily life	5 to <10 cm in diameter or 25 to <100 cm^2 in area or affecting daily life	Diameter ≥10 cm or area ≥100 cm^2 or ulceration or secondary infection or phlebitis or aseptic abscess or wound drainage or seriously affecting daily life	Abscess, exfoliative dermatitis, dermal or deep tissue necrosis
Redness*	Diameter 2.5 to <5 cm or area 6.25 to <25 cm^2 without affecting	5 to <10 cm in diameter or 25 to <100 cm^2 in area or	Diameter ≥10 cm or area ≥100 cm^2 or ulceration or secondary infection or phlebitis or aseptic	Abscess, exfoliative dermatitis,

	or slightly affecting daily life	affecting daily life	abscess or wound drainage or seriously affecting daily life	dermal or deep tissue necrosis
Rash*	Diameter 2.5 to <5 cm or area 6.25 to <25 cm^2 without affecting or slightly affecting daily life	5 to <10 cm in diameter or 25 to <100 cm^2 in area or affecting daily life	Diameter ≥10 cm or area ≥100 cm^2 or ulceration or secondary infection or phlebitis or aseptic abscess or wound drainage or seriously affecting daily life	Abscess, exfoliative dermatitis, dermal or deep tissue necrosis
Pruritus	Itching at injection site, relieved within 48 hours	Itching at injection site, did not alleviate within 48 h after treatment	Affecting daily life	..

* The maximum measured diameter or area should be used for induration and swelling, rash and redness; evaluation and grading should be based on functional grade and actual measurement results, and higher grading indicators should be selected.

Table S3. Severity grading criteria for systemic adverse events and vital signs

	Grade 1	Grade 2	Grade 3	Grade 4
Diarrhea	Mild or transient, 3-4 times/day, abnormal stool, or mild diarrhea lasting less than 1 week	Moderate or persistent, 5-7 times/day, abnormal stool, or diarrhea >1 week	>7 times/day, abnormal stool, or hemorrhagic diarrhea, orthostatic hypotension, electrolyte imbalance, requiring intravenous infusion >2 L	Hypotensive shock, hospitalization
Decreased appetite	Decreased appetite, not affecting food intake	Decreased appetite, reduced food intake, not affecting body weight	Decreased appetite, and significantly reduced body weight	Need intervention (such as gastric tube feeding, parenteral nutrition)
Vomiting	1-2 times/24 hours without	3-5 times/24 hours or affecting activity	>6 times within 24 hours or requiring	Hospitalization or other nutrition

	affecting activity		intravenous fluid infusion	routes due to hypotensive shock
Nausea	Transient (<24 hours) or intermittent and basically normal food intake	Persistent nausea leads to reduced food intake (24-48 hours)	Persistent nausea leads to almost no food intake (>48 hours) or requires intravenous fluids	Life threatening (e.g., hypotensive shock)
Muscle pain (non-inoculated site)	Does not affect daily activities	Slightly affects daily activities	Severe muscle pain, seriously affects daily activities	Emergency or hospitalization
Joint pain	Mild pain, not affecting daily activities	Moderate pain, requiring analgesics and/or pain interferes with functioning, yet not affecting daily activities	Severe pain, seriously affecting daily activities	Emergency or hospitalization

Headache	Not affecting daily activities, no treatment required	Transient, slightly affecting daily activities, may need treatment or intervention	Seriously affecting daily activities, need treatment or intervention	Intractability, need emergency or hospitalization
Cough	Transient, no treatment required	Persistent cough, effective treatment	Paroxysmal cough, uncontrolled treatment	Emergency or hospitalization
Fatigue	Normal activity is weakened <48 hours, without affecting the activity	Normal activity is weakened by 20%~50% >48 hours, slightly affecting the activity	Normal activity is weakened by >50%, seriously affecting daily activities, unable to work	Unable to take care of oneself, emergency or hospitalization
Pruritus	Mild or localized; topical intervention indicated	Widespread and intermittent; skin changes from scratching (e.g., edema, papulation,	Widespread and constant; limiting self-care activities of daily living or sleep; systemic corticosteroid or	-

		excoriations, lichenification, oozing/crusts); oral intervention indicated; limiting instrumental activities of daily living	immunosuppressive therapy indicated	
Skin rash (exanthema)†	Present, but asymptomatic	Symptomatic (pruritus/pain), but interferes only slightly with daily activities	Symptomatic, prevents daily activities	Emergency or hospitalization
Allergic reaction	Systemic intervention not indicated	Oral intervention indicated	Bronchospasm; hospitalization indicated for clinical sequelae; intravenous intervention indicated	Life-threatening consequences; urgent intervention indicated
Vital signs	-	-	-	-

Fever (oral temperature)	37.5～＜ 38.2^{0}C	38.2～＜38.7^{0}C	≥38.7^{0}C	≥39.7^{0}C, lasting more than 3 days

† Specify if the skin rash is located in any specific body part or if it is widespread.

The severity of the unsolicited clinical adverse events was classified through a numeric scale of one to five, which was created based on the grading depicted in Table S4.

Table S4. Severity grading criteria for unsolicited adverse events

Grade 1 (Mild)	Transient (<48 hours) or mild discomfort; no medical intervention/therapy required
Grade 2 (Moderate)	Mild to moderate limitation in activity - some assistance may be needed; no or minimal medical intervention/therapy required
Grade 3 (Severe)	Marked limitation in activity, some assistance usually required; medical intervention/therapy required, hospitalizations possible
Grade 4 (Life-threatening)	Extreme limitation in activity, significant assistance required; significant medical intervention/therapy required, hospitalization or hospice care probable
Grade 5	Death
