# Supplementary material for: Efficacy, Immunogenicity, and Safety of the Two-Dose Schedules of TURKOVAC versus CoronaVac in Healthy Subjects: A Randomized, Observer-Blinded, Non-Inferiority Phase III Trial
Source: Vaccines (Basel). 2022 Nov 4;10(11):1865. doi: 10.3390/vaccines10111865 (PMC9698857; doi:10.3390/vaccines10111865)
Supplement: Supplementary file 1 [file vaccines-10-01865-s001.zip › Supplementary Material S4.pdf]

Supplementary Material S4. List of participating sites

Centers	Number of volunteers recruited
Hacettepe University Faculty of Medicine Adult Hospital, Department of Internal Medicine, Division of General Internal Medicine, Ankara, Türkiye	208
Ankara City Hospital Infectious Diseases and Clinical Microbiology Clinic, Ankara, Türkiye	304
University of Health Sciences, Izmir Tepecik Training and Research Hospital, Infectious Diseases Clinic, Izmir, Türkiye	22
Erciyes University Faculty of Medicine, Department of Infectious Diseases and Clinical Microbiology, Kayseri, Türkiye	164
Kocaeli University Faculty of Medicine, Department of Infectious Diseases and Clinical Microbiology, Kocaeli, Türkiye	14
Başakşehir Çam and Sakura City Hospital, Infectious Diseases and Clinical Microbiology Clinic, Istanbul, Türkiye	456
Kayseri City Training and Research Hospital, Department of Infectious Diseases and Clinical Microbiology, Kayseri, Türkiye	112

Uludağ University Health Application and Research Center, Department of Infectious Diseases and Clinical Microbiology, Bursa, Türkiye	10
**Total**	**1290**
