# Supplementary material for: Efficacy, Immunogenicity, and Safety of the Two-Dose Schedules of TURKOVAC versus CoronaVac in Healthy Subjects: A Randomized, Observer-Blinded, Non-Inferiority Phase III Trial
Source: Vaccines (Basel). 2022 Nov 4;10(11):1865. doi: 10.3390/vaccines10111865 (PMC9698857; doi:10.3390/vaccines10111865)
Supplement: Supplementary file 1 [file vaccines-10-01865-s001.zip › Supplementary Material S5.pdf]

Supplementary Material S5. Distribution of COVID-19 cases with regard to the WHO Clinical Progression Scale

Table S5. COVID-19 severity and symptoms of the cases in the efficacy analysis with regard to WHO Clinical Progression Scale (the highest score during the course of the disease is given here)

**Patient state**	**Descriptor**	**CoronaVac arm n (%)**	**TURKOVAC arm n (%)**	**Total n (%)**
Ambulatory mild disease	2 Symptomatic- independent	60 (13.1)	35 (7.7)	95 (10.4)
	3 Symptomatic- assistance needed	0 (0)	0 (0)	0 (0)
Hospitalized: moderate disease	4 Hospitalized (If hospitalization is for isolation only record the status with an outpatient)- no oxygen therapy	0 (0)	0 (0)	0 (0)
	5 Hospitalized; oxygen by mask or nasal prongs	1 (0.2)	0 (0)	1 (0.1)
Hospitalized: severe diseases	6 Hospitalized-oxygen by non-invasive or high flow ventilation	0 (0)	0 (0)	0 (0)

Total	61 (13.3)	35 (7.7)	96 (10.5)
