# Supplementary material for: Efficacy, Immunogenicity, and Safety of the Two-Dose Schedules of TURKOVAC versus CoronaVac in Healthy Subjects: A Randomized, Observer-Blinded, Non-Inferiority Phase III Trial
Source: Vaccines (Basel). 2022 Nov 4;10(11):1865. doi: 10.3390/vaccines10111865 (PMC9698857; doi:10.3390/vaccines10111865)
Supplement: Supplementary file 1 [file vaccines-10-01865-s001.zip › Supplementary Material S6.pdf]

Supplementary Material S6. Adverse events

Table S6. Distribution of adverse events

	**CoronaVac arm (n=459) N=459**		**TURKOVAC arm (n=456) N=456**		**Total (n=915) N=915**		
	Events n	Subjects n (%)	Events n	Subjects n (%)	Events n	Subjects n (%)	p
Total AEs	754	228 (49.7)	896	268 (58.8)	1650	496 (54.2)	0.006
Solicited AEs	700	225 (49)	840	267 (58.6)	1540	492 (53.8)	0.004
Unsolicited AEs	54	45 (9.8)	56	45 (9.9)	110	90 (9.8)	0.974
Systemic AEs	636	186 (40.5)	625	178 (39)	1261	364 (39.8)	0.646
Local AEs	118	98 (21.4)	271	199 (43.6)	389	297 (32.5)	<0.001
Grade 1 AEs	632	207 (45.1)	806	256 (56.1)	1438	463 (50.6)	0.001
Grade 2 AEs	121	52 (11.3)	90	37 (8.1)	211	89 (9.7)	0.101
Grade 3 AEs	1	1 (0.2)	0	0 (0)	1	1 (0.1)	1
Grade 4 AEs	-	-	-	-	-	-	-

AEs, Adverse events.

Table S7. Distribution of serious adverse events

Vaccination arm	Definition of Serious Adverse Events	Date of Hospitalization	Follow-up	Date of Start	Date of 1st dose vaccination	Date of 2nd dose vaccination
CoronaVac	Hospitalization for kidney stones	04/08/2021	Yes	04/08/2021	05/07/2021	06/08/2021
TURKOVAC	Hospitalization for febrile COVID-19	12/08/2021	Yes	12/08/2021	08/07/2021	05/08/2021
TURKOVAC	Hospitalization for COVID-19	19/09/2021	Yes	19/09/2021	08/09/2021	18/01/2022
TURKOVAC	Hospitalization for febrile COVID-19	02/10/2021	Yes	02/10/2021	26/08/2021	23/09/2021
CoronaVac	Hospitalization for COVID-19	19/12/2021	Yes	19/12/2021	06/09/2021	04/10/2021
CoronaVac	Diabetes Mellitus	24/01/2022	Yes	24/01/2022	23/11/2021	30/12/2021
CoronaVac	Nasal Septal Deviation	19/02/2022	No	19/02/2022	8/11/2021	06/12/2021
