# Supplementary material for: Efficacy, Immunogenicity, and Safety of the Two-Dose Schedules of TURKOVAC versus CoronaVac in Healthy Subjects: A Randomized, Observer-Blinded, Non-Inferiority Phase III Trial
Source: Vaccines (Basel). 2022 Nov 4;10(11):1865. doi: 10.3390/vaccines10111865 (PMC9698857; doi:10.3390/vaccines10111865)
Supplement: Supplementary file 1 [file vaccines-10-01865-s001.zip › Supplementary Material S7.pdf]

Supplementary Material S7. TURKOVAC Study Group

Dr. Alper Gunduz, Dr. Onur Ozalp, and Dr. Soner Sabirli from Department of Infectious Diseases and Clinical Microbiology, University of Health Sciences, Başaksehir Cam and Sakura City Hospital, 34480 Istanbul, Türkiye

Assoc. Prof. Bircan Kayaaslan, Assoc. Prof. Imran Hasanoglu, and Assist. Prof Ayşe Kaya Kalem from Infectious Diseases and Clinical Microbiology Clinic, Ankara Yildirim Beyazit University, Ankara City Hospital, 06800 Ankara, Türkiye

Dr. Muge Ayhan, Dr. Belgin Coskun, Dr. Omer Aydos, and Dr. Gamze Kaya from Infectious Diseases and Clinical Microbiology Clinic, Ankara City Hospital, 06800 Ankara, Türkiye

Assoc. Prof. Dr. Ahmet Cagkan Inkaya and Dr. Ahmet Gorkem Er from Department of Infectious Diseases and Clinical Microbiology, Hacettepe University Faculty of Medicine, 06230 Ankara, Türkiye

Assist. Prof. Zeynep Ture Yuce and Assist Prof. Gamze Kalin Unuvar from Department of Infectious Diseases and Clinical Microbiology, Erciyes University Faculty of Medicine, 38030 Kayseri, Türkiye

Dr. Aysin Kilinç Toker from Department of Infectious Diseases and Clinical Microbiology, Kayseri City Training and Research Hospital, 38080 Kayseri, Türkiye

Dr. Omer Demir from Infectious Diseases Clinic, University of Health Sciences, Izmir Tepecik Training and Research Hospital, 35020 Izmir, Türkiye

Dr. Sonay Arslan and Dr. Zeynep Bayraktar from Department of Infectious Diseases and Clinical Microbiology, Kocaeli University Faculty of Medicine, 41001 Kocaeli, Türkiye

Dr. Ugur Onal from Department of Infectious Diseases and Clinical Microbiology, Bursa Uludag University Faculty of Medicine, 16059 Bursa, Türkiye
